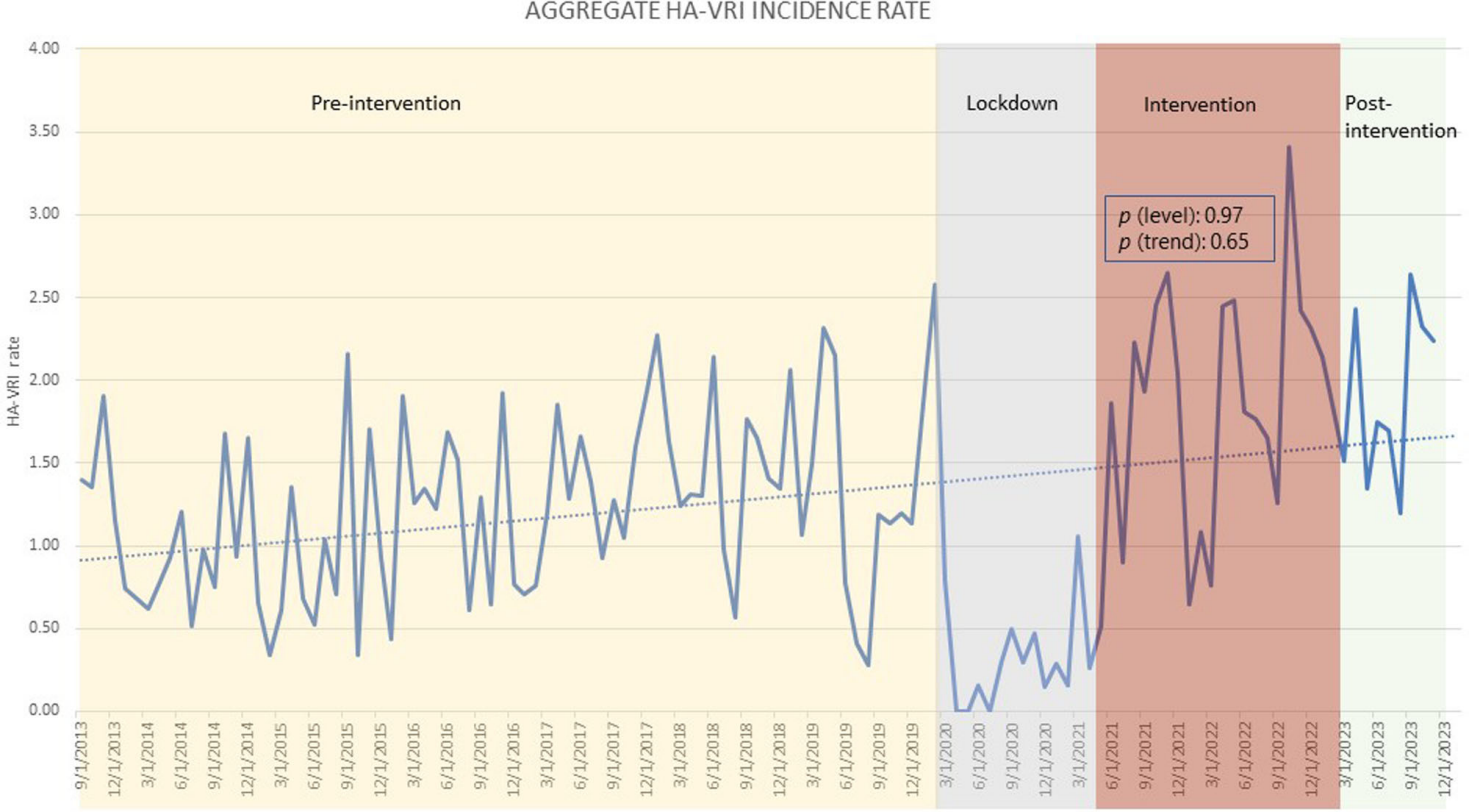# Impact of Universal Masking by Healthcare Personnel on Non-SARS-CoV-2 Healthcare-Associated Viral Respiratory Infections

**DOI:** 10.1017/ash.2024.313

**Published:** 2024-09-16

**Authors:** Ayelet Rosenthal, Riley Moore, Joseph Fishbein, Larry Kociolek

**Affiliations:** Lurie Children’s Hospital of Chicago, Northwestern University; Lurie Children’s Hospital; Ann & Robert H. Lurie Childrens Hospital of Chicago

## Abstract

**Background:** The impact of universal masking on transmission of endemic respiratory viruses in children’s hospitals is unknown. Our objective was to measure the association between universal masking by healthcare personnel and the incidence of non-SARS-CoV-2 healthcare-associated viral respiratory infections (HA-VRIs) in a free-standing academic pediatric medical center during the COVID-19 pandemic. **Methods:** In this quasi-experimental study, we measured the incidence rate of non-SARS-CoV-2 HA-VRIs (VRI diagnosed on or after hospital day 3 by one of several molecular assays) during three time periods: prior to the COVID-19 pandemic (pre-intervention: September 2013 - February 2020); during universal masking (intervention: May 2021 - March 2023); and after universal masking was lifted (post-intervention: April 2023 - November 2023). Although universal masking was implemented in late March 2020, we exclude the lockdown period of strict COVID-19-related public health mitigations (i.e., school closures and shelter-in-place advisories) during which community prevalence of non-SARS-CoV-2 respiratory viruses was minimal in our region (March 2020 to April 2021). By negative binomial regression analysis, we compared the level and trend of HA-VRIs between the pre-intervention and intervention periods. **Results:** Figure 1 illustrates the incidence rate of non-SARS-CoV-2 HA-VRI during the pre-intervention, lockdown, intervention, and post-intervention periods. The aggregate non-SARS-CoV-2 HA-VRI incidence rate during the pre-intervention, intervention, and post-intervention periods was 1.25, 1.84, and 1.96 HA-VRIs per 1000 patient days, respectively. There was no significant difference in the level (p = 0.96) or trend (p = 0.67) of HA-VRI incidence rate between the pre-intervention and intervention periods. **Conclusion:** Universal masking was not associated with a decrease in the incidence rate of non-SARS-CoV-2 HA-VRIs at our children’s hospital during the COVID-19 pandemic. These findings suggest that universal masking may not be an effective infection prevention measure in children’s hospitals during periods of increased endemic respiratory viral transmission in the community.

**Disclosure:** Larry Kociolek: Research Support - Merck

**Figure:**